# Integrated single-cell and bulk transcriptomic analyses reveal a stem-like epithelial subpopulation in adenocarcinoma of the esophagogastric junction and identify VASN as a novel regulator of tumor stemness

**DOI:** 10.3389/fimmu.2026.1817030

**Published:** 2026-05-13

**Authors:** Hang Zheng, Shuaibo Wang, Heshu Liu, Jing Wang, Bin Hu

**Affiliations:** 1 Department of Thoracic Surgery, Beijing Institute of Respiratory Medicine and Beijing Chao-Yang Hospital, Capital Medical University, Beijing, China; 2 Cancer Center, Beijing Tongren Hospital, Capital Medical University, Beijing, China

**Keywords:** adenocarcinoma of esophagogastric junction, immunological niche, precision oncology, tumor stemness, VASN

## Abstract

**Background:**

Cancer stem cells (CSCs) are widely recognized as key contributors to tumorigenesis, progression, therapeutic resistance and immune evasion in adenocarcinoma of esophagogastric junction (AEG). Nevertheless, the stem-like epithelial subpopulations that may contribute to immunological niche formation remain poorly characterized, hindering the identification of effective therapeutic targets.

**Methods:**

Owing to their ability to delineate cellular heterogeneity, single-cell RNA sequencing (scRNA-seq) and bulk RNA sequencing (bulk RNA-seq) analyses were employed to characterize the CSCs-like subpopulation in AEG tumor tissues. Utilizing scRNA and bulk transcriptomics cohorts, this study systematically identified the putative CSC-like malignant epithelial subpopulation, and a series of bioinformatics and machine learning as well as *in vitro* functional experiments further verified VASN on AEG malignancy.

**Results:**

Six main cell types were identified in AEG tumors, and six clusters of malignant epithelial cells were filtered out via inferCNV analysis. The cluster 2 (C2) subgroup exhibited pronounced elevation of CytoTRACE, epithelial-mesenchymal transition, Wnt signaling and stem cell differentiation scores, trajectory analysis positioned C2 subgroup at the initiation stage of pseudotime, which comprehensively identified C2 as stem-like neoplastic subpopulation. Through the integration of high dimensional weighted gene co-expression network analysis (hdWGCNA), Branch Expression Analysis Modeling (BEAM) and differentially expressed genes, 21 genes were identified as hub genes of C2 cluster. The CSC-related prognostic model was developed via LASSO Cox regression analysis. Random survival forest further identified VASN as candidate CSCs marker of AEG. Additionally, knock down of VASN in AEG cell line reduced migration and invasion abilities as well as CSCs markers expressions. Mechanistically, *in vitro* and bioinformatics analysis revealed a potential link between VASN and stemness-associated Wnt/β-catenin signaling.

**Conclusions:**

Our investigation identified a stem-like epithelial subpopulation and established a stem-related gene signature, offering insights into stemness-associated tumor progression and immunological niche dynamics in AEG. Furthermore, we identified VASN as a putative regulator of malignant stem phenotypes that may influence tumor microenvironment remodeling.

## Introduction

Gastric adenocarcinoma (GA) remains a global health challenge, ranking fifth in cancer-related mortality worldwide (1). Anatomically, GA could be classified into cardia tumors arising at the esophagogastric junction area, and non-cardia tumors located in the more distal pyloroantral region of the stomach. While the incidence of non-cardia GA has declined over recent decades, cardia GA (also known as adenocarcinoma of esophagogastric junction, AEG) has seen a seven-fold increase in prevalence, and China accounts for 70% of new cardia GA cases and 50% of non-cardia GA cases, respectively (2, 3). Due to the lack of early symptoms, a substantial proportion of patients with AEG are diagnosed at an advanced or metastatic stage, which contributes to compromised survival outcomes, especially among Asian populations (3).

Cancer stem cells (CSCs), a small yet critical neoplastic subpopulation, are characterized as key drivers for tumor initiation, metastasis, and therapeutic resistance (4, 5). Increasing evidence also suggests that CSC-associated programs contribute to immune evasion and influence the organization of the tumor microenvironment. Conventional anti-cancer therapies primarily target the proliferating bulk population of non-CSCs within a tumor, thereby leaving CSCs to persist and propagate, ultimately leading to treatment failure and poor clinical outcomes (6). CSCs therefore represent a central challenge in overcoming resistance across solid malignancies. Despite extensive advances in characterizing CSCs markers in gastric and esophageal cancers, research specifically focused on AEG remains scarce (7). Given the emerging evidence that AEG should be regarded as a distinct disease entity from gastric or esophageal cancers (8, 9), there is an urgent need to elucidate the specific CSC-associated signatures and molecular characteristics that might contribute to tumor progression and microenvironment remodeling in AEG.

Single-cell RNA sequencing (scRNA-seq) has revolutionized cellular phenotyping by enabling high-resolution dissection of transcriptional heterogeneity at single-cell resolution. This level of resolution cannot be achieved using conventional bulk RNA sequencing, which only yields averaged transcriptomic profiles across cell populations (10–12). scRNA-seq dissects cellular heterogeneity at single-cell resolution, offering unbiased, high-throughput insights into rare cell subsets, gene regulatory networks, and differentiation trajectories (13). This technology has become the gold standard in stem cell biology, particularly for mapping differentiation trajectories, reconstructing gene regulatory networks, and unraveling the complex transcriptomic landscapes of tumor ecosystems, where diverse cell types express unique molecular signatures during oncogenesis, thereby enabling the identification of multipotent progenitor cells and the reconstruction of differentiation trajectories along potency gradients (14, 15).

In this study, we employed integrated scRNA-seq and bulk RNA sequencing analyses to systematically characterize cellular heterogeneity of AEG epithelial cells, and a distinct putative CSC-like malignant epithelial subpopulation with unique transcriptional characteristics was identified. By applying differentially expressed gene analysis (DEG), high dimensional weighted gene co-expression network analysis (hdWGCNA), differentiation trajectory mapping and random survival forest modeling, we constructed a stemness-associated gene signature and pinpointed VASN as a central hub gene governing stem-like properties in AEG. Experimental validation further confirmed the functions and underlying mechanisms of VASN in AEG stemness. Given the imperative need to elucidate the intricate molecular mechanisms of AEG stemness for the development of more effective therapeutic strategies, our findings may provide novel insights into target identification and precision medicine for AEG patients.

## Materials and methods

### scRNA-seq data collection and detailed single-cell analysis pipeline

We retrieved the scRNA-seq dataset GSE183904 (16) from the Gene Expression Omnibus (GEO, http://www.ncbi.nlm.nih.gov/geo/) database, which comprises two AEG tumor samples (Location: Cardia) (GSM5573479 and GSM5573482) for subsequent bioinformatics analysis. The Seurat R package (version 4.3.0) (17) was applied for modular downstream scRNA-seq analysis. The “CreateSeuratObject” function was firstly utilized to create the Seurat object. To ensure data quality, cells demonstrating suboptimal sequencing metrics were systematically removed. Specifically, cells expressing fewer than 500 genes or more than 6,000 genes (potential empty droplets or doublets), or having over 20% mitochondrial transcripts were excluded from the analysis. The filtered dataset consisted of 13,409 cells, which was then normalized with LogNormalize method using the “NormalizeData” function. The top 2,000 genes exhibiting significant variability were identified via “FindVariableFeatures” function, and expression values were scaled using “ScaleData” function prior to principal component analysis (PCA). Subsequently, the “harmony” R package (version 0.1.0) was employed to correct batch effects using the Harmony algorithm (18). Afterwards, we applied “FindNeighbors” and “FindCluster” functions based on the top 20 identified principal components for cell clustering (resolution = 0.5), and dimensionality visualized the results through t-distributed stochastic neighbor embedding (t-SNE) by using “RunTSNE” function. t−SNE was used for visualization due to the small dataset size and its strength in preserving local neighbor relationships, which aided in separating the putative CSC−like cluster. Cluster-specific marker genes were then identified using “FindAllMarkers” function, with parameters configured as test.use=“wilcox”, min.pct = 0.25, and logfc.threshold > 0.25. Finally, CellMarker database (http://xteam.xbio.top/CellMarker/) (19) was utilized to manually annotate the cells, and transcriptomic distributions were visualized using the ggplot2-based “FeaturePlot” and “DotPlot” functions.

### Malignant AEG epithelial cells identification and reanalysis

Epithelial cells from AEG tumors were extracted and analyzed following the established experimental steps. To distinguish between malignant cells and non-malignant AEG epithelial cells, the InferCNV (version 1.18.0) R package was implemented for copy number variation (CNV) profiling. This approach employs a reference-based normalization strategy, wherein transcriptional intensities of epithelial cells were subjected to systematically comparative analysis against the reference endothelial cells across genomic positions. Cells with CNV score > 0.001 and CNV correlation > 0.4 were designated as malignant epithelial cells (20), which were extracted and subsequently reanalyzed with a resolution of 0.3 for graph-based clustering.

### Stemness and pseudotime analysis

CytoTRACE algorithm was utilized to elucidate the differential degrees of differentiation among distinct malignant AEG epithelial subgroups. By calculating the CytoTRACE score, we were able to deduce their individual differentiation states (21). Single-cell trajectory inference was conducted using Monocle2 algorithm (22) with “DDRTree” dimension reduction and default parameter configurations. To delineate lineage commitment dynamics, Branch Expression Analysis Modeling (BEAM) analysis was carried out for evaluation of branch point-specific genes along reconstructed pseudotemporal trajectories. Cellular differentiation trajectories across malignant AEG epithelial subpopulations were also inferred through the Slingshot algorithm (23) implemented in the SCP R package (version 0.5.1) via the “RunSlingshot” function (https://github.com/zhanghao-njmu/SCP).

### Differential expression patterns and functional enrichment analysis

Differentially expressed cluster-specific genes were identified through an analytical pipeline incorporating two complementary approaches: (1) multi-group comparison using the “FindAllMarkers” function and (2) pairwise analysis implemented via the “FindMarkers” function within the Seurat computational framework. Genes with P values less than 0.05 and average fold-change greater than 1 were regarded as significant differentially expressed markers. Subsequently, these markers were subjected to Gene Ontology (GO) enrichment analyses through clusterProfiler R package (24), and an adjusted p-value of less than 0.05 was set for enrichment significance. In addition, functional characterization of epithelial subpopulations was also performed through Gene Set Enrichment Analysis (GSEA), utilizing the c5.go.bp.v7.5.1.symbols gene set collection from the Molecular Signatures Database (MSigDB) (25) for biological process annotation.

### Gene set variation analysis and scoring

Cellular pathway activity was quantified through Gene Set Variation Analysis (GSVA) (26), which was conducted based on Gene Ontology Biological Processes (GO-BP) and hallmark pathway gene sets obtained from MSigDB. Pathway activity differences were quantified through comparative analysis of enrichment scores across distinct epithelial subpopulations. In addition, we also applied AUCell method to quantify the activity scores of MSigDB-curated pathway gene sets in distinct epithelial subpopulations.

### Transcription factor program analysis

Transcriptional factors (TFs) and regulatory networks within each malignant AEG epithelial subgroup were comprehensively assessed using pySCENIC (version 0.11.0) algorithm within Python 3.9 environment (27). Firstly, GRNBoost algorithm was employed to predict co-expression modules integrating TFs and target genes. Subsequently, RcisTarget-driven DNA motif enrichment analysis was carried out to identify direct binding targets of TFs (regulons). Next, AUCell algorithm was used for regulon activity quantifications, and the top regulons with the highest scores in each subpopulation were evaluated.

### Cell-cell communication analysis

We applied the CellChat R package (v1.6.1) (28) to delineate cell–cell signaling crosstalk within the tumor microenvironment. Overexpressed ligands and receptors in each cell cluster were initially detected via the “identifyOverExpressedGenes” and “identifyOverExpressedInteractions” functions. The probabilities of intercellular interactions were then calculated with the “computeCommunProb” function.

### HdWGCNA

HdWGCNA was implemented using the hdWGCNA R package (version 0.2.26) to establish scale-free topological networks at single-cell resolution (29). The analytical workflow initiated with metacell matrix transposition via k-nearest neighbors (KNN) algorithm by “MetacellsByGroups” function. For the scale-free topological model, the optimal soft-power was set as 9 with R² > 0.8. Subsequently, co-expression networks were built via ‘ConstructNetwork’ function. Module eigengenes were retrieved through ‘ModuleEigengenes’ function, and the kME values were then calculated using the ‘ModuleConnectivity’ function. Genes with relatively higher kME values were more likely to be the hub genes. The correlation between modules and AEG epithelial traits was computed via the ‘ModuleTraitCorrelation’ function and shown through bubble plots and heatmap.

To identify stemness−associated genes, we took the intersection of three gene sets: (1) genes from the hdWGCNA module most correlated with the putative CSC−like cluster; (2) genes identified as branch−dependent by BEAM analysis; and (3) DEGs between the putative CSC−like cluster and other epithelial clusters.

### Bulk RNA-seq data collection and pre-processing

Two bulk RNA-seq and clinical prognostic data of gastric cancer (GC) [TCGA-STAD and GSE84437 (30)] cohorts were respectively downloaded from the UCSC Xena (https://xenabrowser.net/datapages/) (31) and GEO database, which collectively included 330 (including 79 AEG) GC samples in TCGA-STAD, and 431 GC samples in GSE84437. For TCGA-STAD, fragments per kilobase per million mapped reads (FPKM) standardized data were transformed to transcripts per million (TPM) values to guarantee data consistency and comparability (32). For GSE84437, the robust multiarray averaging (RMA) algorithm was implemented for preprocessing.

### Prognostic model construction and validation

Candidate hub genes were put into LASSO Cox regression analysis on TCGA-AEG samples (training dataset) for overall survival (OS)-related prognostic genes screening and model construction via the cv.glmnet function of glmnet R package. Ten-fold cross-validation (10-fold CV) was applied with 1000 maximum iterations (maxit = 1000), and the optimal penalty parameter λ was selected as λ.min. The stemness risk model was subsequently constructed by integrating the multiplication of standardized gene expression values and its LASSO coefficients (βi × Expi). The entire TCGA-STAD and GSE84437 cohorts were used as external cohorts for the prognostic model validations. Patients were stratified into high and low stemness risk subgroups by the median risk score as cutoff. By applying OCLR algorithm, mRNA stemness index (mRNAsi) scores were calculated (33), and the correlations between our AEG stemness-related risk score and other published stemness-related model with mRNAsi were further assessed and compared. Finally, the Tumor Immune Dysfunction and Exclusion (TIDE) algorithm was applied to evaluate the potential responsiveness of patients in different stemness risk cohorts to immunotherapy (34), and xCell algorithm was applied for the calculation of immune infiltrations scores using xCell R packsge (35).

### Enrichment analysis

GSEA was performed on hallmark and antigen processing and presentation gene sets from MSigDB using the “ClusterProfiler” R package (24, 25). The TCGA-AEG cohort was stratified into VASN-high and VASN-low-expression subgroups based on the median VASN expression. Subsequent comparisons between these groups were conducted with a significance threshold of p < 0.05.

### Cell culture and treatment

Human AEG cell line OE19 [established in 1993 from type II AEG patient (36)] was purchased from Procell (Wuhan, China) and cultured with RPMI-1640 medium (Thermo Fisher Scientific, Rockford, IL) supplemented with 10% FBS and 1% penicillin-streptomycin (Thermo Fisher Scientific) in an incubator with 5% CO2 at 37 °C. OE19 cells were transfected with 75 pmol of VASN siRNA or negative control siRNA (Thermo Fisher, No. 4392420 and No. AM4611) by Lipofectamine 3000 (Invitrogen, USA). The siRNA-lipid complexes were prepared in 250 μL Opti-MEM I Reduced Serum Medium (Gibco, USA) and then added to the culture medium. Cells were maintained for 48 h post-transfection prior to harvesting for protein extraction.

### Western blotting

The detailed Western blotting (WB) protocol has been described previously (37). In brief, AEG cells were lysed in RIPA buffer (Beyotime, China) supplemented with 1 mM PMSF and protease inhibitor cocktail. Lysates were incubated on ice for 30 min and centrifuged to collect supernatants. Protein concentrations were determined using a BCA assay (Invitrogen, USA). Equal amounts of protein were separated by 10% SDS-PAGE and then electroblotted to PVDF membranes (Millipore, Burlington, MA, USA). After blocking with 5% milk for one hour, the membranes were incubated with primary antibodies overnight at 4 °C, followed by HRP-conjugated secondary antibodies (1:8000, ZSGB-BIO, Beijing, China) for one hour at room temperature. The primary antibodies used were as follows: anti-SOX2 (3579T, CST), anti-CD44 (ab243894, Abcam), anti-OCT4 (11263-1-AP, Proteintech), anti-Nanog (4903T, CST), anti-VASN (MAB2140-SP, R&D systems), anti-CD133 (ab278053, Abcam), anti-β-catenin (ab32572, Abcam), anti-Lamin B1 (ab16408, Abcam), anti-E-cadherin (9782T, CST), anti- N-cadherin (9782T, CST), anti-Snail (9782T, CST), anti-Vimentin (5741T, CST), anti-c-Myc (ab32072, Abcam), anti-β-actin (66009-1-Ig, Proteintech), and anti-GAPDH (60004-1-Ig, Proteintech).

### Migration and invasion assays

For assessment of cellular metastatic potential, 5 × 10^4^ OE19 cells were plated in the upper compartment of Transwell plates (Corning, New York, USA) containing 200μL serum-free medium, while the lower chamber was filled with 10% FBS-supplemented complete medium. After 24-hour incubation, migrated cells were fixed with 4% paraformaldehyde, stained with 0.1% crystal violet, and visualized through an optical microscope. For invasion assays, the upper chamber was pre-coated with Matrigel (Corning, New York, USA). OE19 cells were then seeded onto the upper chamber in 200 μL serum-free medium, while 700 μL of complete medium with 10% FBS was added to the lower chamber. After 24 hours, invasive cells traversing the matrix barrier were identically fixed, stained, and imaged.

### TOP/FOP luciferase assay

The TOP/FOP Flash luciferase reporter assay was performed to evaluate the effect of VASN knockdown on β-catenin activity. OE19 cells treated with si-NC or si-VASN were co-transfected with TOP/Flash (TCF/LEF transcriptional activity detection) or FOP/Flash (negative control) (Beyotime, China) and cultured for 48h. Luciferase activity was measured using the Dual-Luciferase Reporter Assay Kit (Beyotime, China) according to the manufacturer’s instructions.

### Statistical analysis

Statistical analyses were performed using R software (version 4.3.0). Wilcoxon and Kruskal-Wallis tests were applied for comparisons between two and more than two groups. The Spearman’s correlations analysis was performed. Significance levels were denoted as follows: * for P < 0.05, ** for P < 0.01, *** for P < 0.001, and **** for P < 0.0001. Survival analysis of candidate genes was conducted through the survival (version 3.5-5) and survminer (version 0.4.9) R packages with log-rank test. A two-tailed p-value below 0.05 was considered statistically significant.

## Results

### Single-cell landscape and cell type identification in AEG cancer

**Figure 1:**
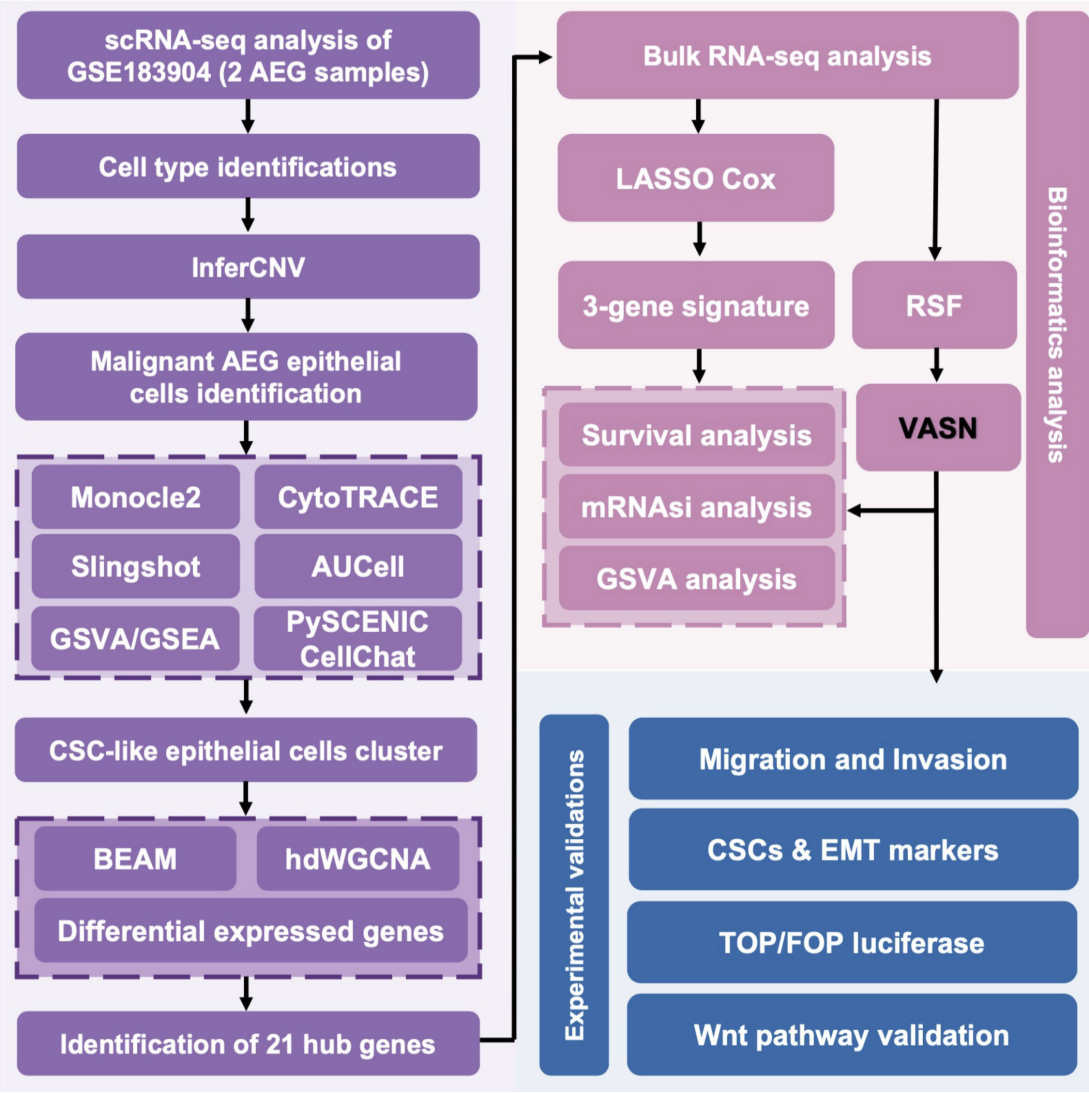
The overall workflow employed in this research.

**Figure 2:**
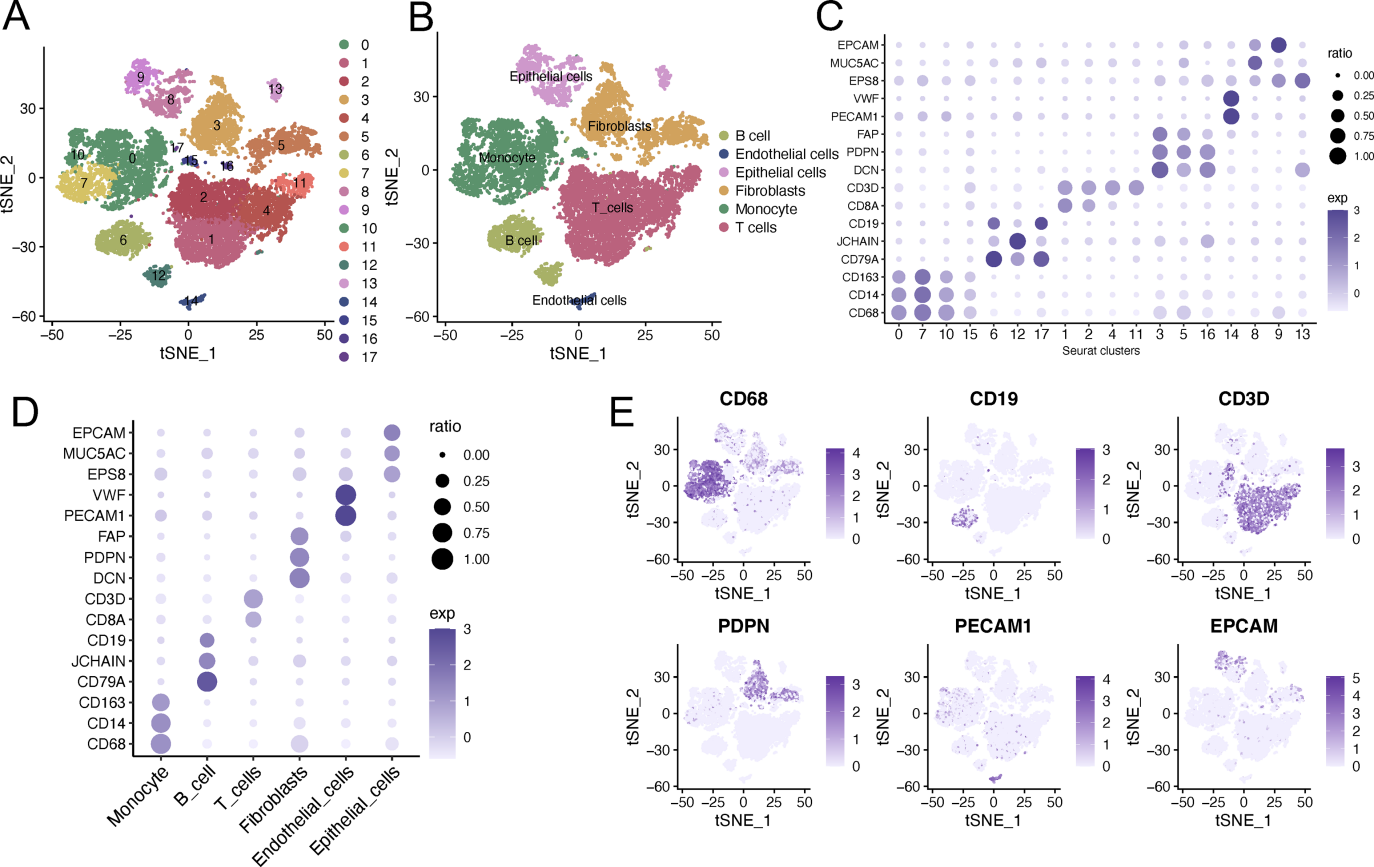
Single−cell transcriptomic landscape of two AEG tumors (13,490 cells). **(A)** tSNE visualization to display the distribution of eighteen distinct seurat clusters in AEG tumor tissues. **(B)** tSNE visualization to display the distribution of six cell types in AEG tumor tissues. **(C, D)** Dot plot illustrating the expression profiles of marker genes in distinct cell clusters and cell types. **(E)** tSNE visualization to display the distribution of six main cell types in AEG tumor tissues. tSNE, t-distributed Stochastic Neighbor Embedding; AEG, Adenocarcinoma of esophagogastric junction.

The study flowchart is depicted in **Figure 1**. The overall bioinformatics analytical workflow consisted of three sequential steps. Firstly, at the single−cell level, we used pseudotime trajectory, CytoTRACE, and pathway enrichment (GSVA, GSEA) to identify a putative CSC−like subpopulation. Second, to identify stemness−associated regulators, we applied three independent algorithms (hdWGCNA, BEAM, and DEG) on single−cell data and took the intersection of their outputs to obtain a robust gene set. Third, these candidate genes were evaluated in bulk RNA−seq cohorts using LASSO Cox regression and random survival forest for further prognostic feature selection. Single-cell transcriptomic profiling was analyzed on two AEG specimens derived from GEO database. Following rigorous quality control and normalization, 13,409 high-quality cells were retained for downstream analysis. These cells were subsequently categorized into 18 distinct clusters and visualized via t-SNE dimensionality reduction plots (**Figure 2A**). Six major cell types were identified and annotated, with their distributions illustrated through dot plots and t-SNE plots (**Figure 2B**), including 1,227 B cells (marker genes were CD19, JCHAIN and CD79A), 167 endothelial cells (marker genes were VWF and PECAM1), 1,409 epithelial cells (marker genes were EPCAM, MUC5AC and EPS8), 2,607 fibroblasts (marker genes were FAP, DCN and PDPN), 3,373 monocytes (marker genes were CD163, CD14 and CD68) and 4,626 T cells (marker genes were CD3D and CD8A) (**Figures 2C–E**).

### Identification and examination of malignant epithelial cell subpopulations

**Figure 3:**
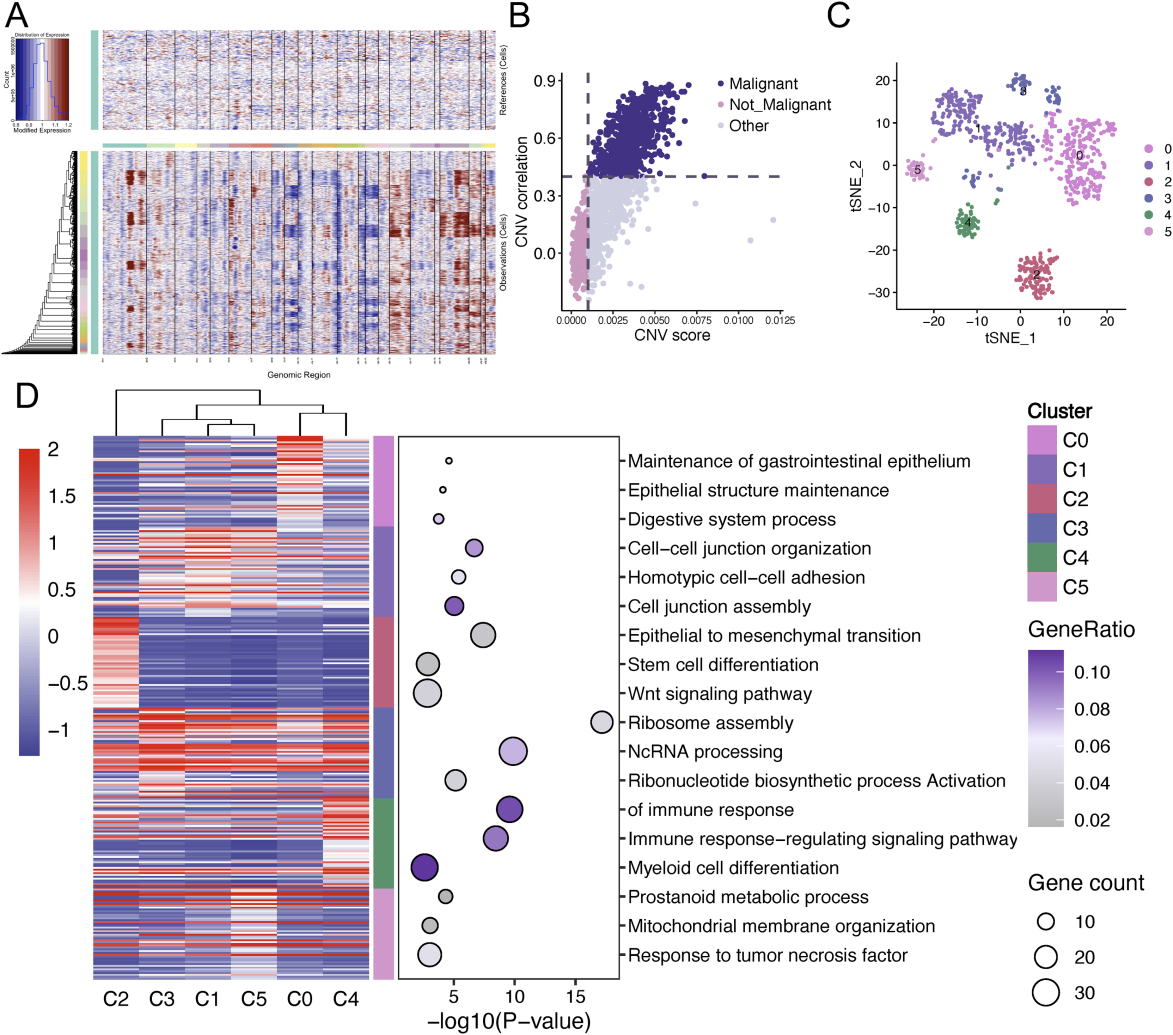
Identification of malignant epithelial cells via CNV analysis. **(A)** CNV heatmap of scRNA-seq data, with endothelial populations serving as the reference. chromosomal deletions were encoded in gradient blue, while amplifications were encoded in progressive red. **(B)** Scatter plot showing the CNV score and CNV correlation of the cells. Malignant cell populations were screened out and highlighted in purple. **(C)** tSNE visualization to display the distribution of six distinct seurat clusters of malignant AEG epithelial cells (n=797). **(D)** Heatmap and dot plot displaying the differentially expressed genes and corresponding Gene Ontology enrichment terms across six separate cell populations. CNV, Copy number variation; tSNE, t-distributed Stochastic Neighbor Embedding; AEG, Adenocarcinoma of esophagogastric junction.

In order to discriminate between malignant and non-malignant cells within the AEG epithelial cell population, we employed InferCNV algorithm (endothelial cells as control) and defined epithelial cells with high-level CNV score and CNV correlation as tumor cells (**Figures 3A, B**). Following filtering, 797 malignant cells were isolated from 1,409 epithelial cells for downstream analysis. Dimensionality reduction and clustering analysis categorized these 797 cells into six seurat clusters with a visualization using t-SNE plots (**Figure 3C**). An integrated view of marker expression profiles and biological functional processes was depicted in **Figure 3D**, revealing that AEG malignant epithelial cluster 2 (C2) demonstrated significant enrichment in epithelial-mesenchymal transition (EMT; p = 5.47e-09), stem cell differentiation (p = 1.37e-03) and Wnt/β-catenin signaling (p = 1.48e-03). These findings suggest that C2 subpopulation might represent a putative CSC-like subpopulation within the AEG malignant epithelial compartment.

### Identification of malignant AEG epithelial C2 cell cluster as putative CSCs

**Figure 4:**
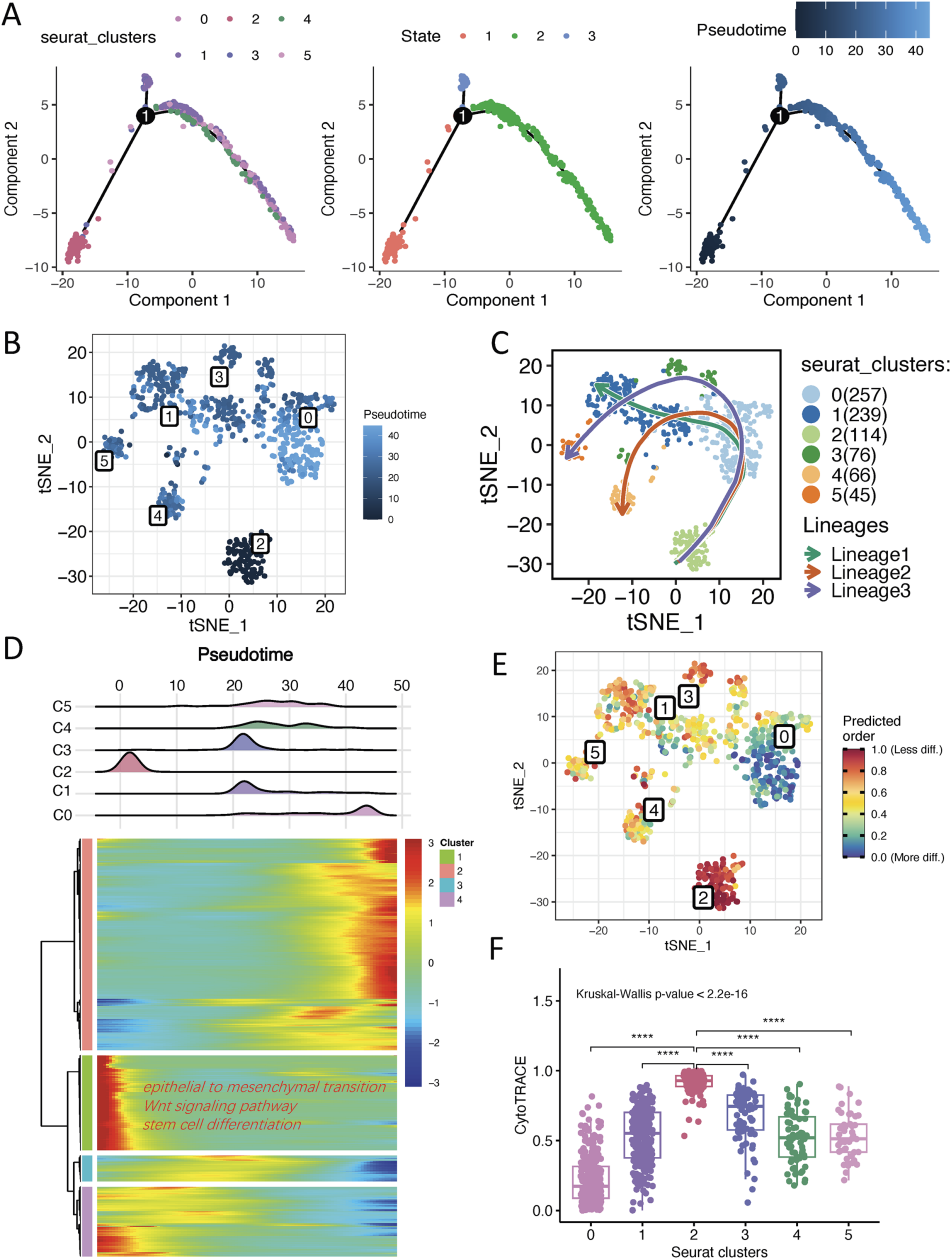
Pseudotime trajectory and stemness inference of malignant epithelial cells. **(A)** The pseudotime trajectories of malignant AEG epithelial cells reveal distinct cellular cluster developmental paths, states and trajectories. **(B)** tSNE plot depicting the monocle2 pseudotime analysis outcomes for cell subsets. **(C)** tSNE plot depicting the slingshot pseudotime analysis outcomes for cell subsets. **(D)** Heatmap illustrating the dynamics of pseudotime−associated genes expressions over pseudotime and the terms of GO-BP enrichment analysis of genes at pseudotime origin state. Upper ridge plot displays cell density distribution of subclusters C0–C5 along pseudotime (shared x−axis). **(E)** tSNE plot depicting the CytoTRACE scores among cell subsets. **(F)** Box plots for the CytoTRACE scores among cell subsets. ****p < 0.0001. AEG, Adenocarcinoma of esophagogastric junction; tSNE, t-distributed Stochastic Neighbor Embedding; DEG, Differentially Expressed Gene, GO-BP, Gene Ontology Biological Processes.

Following additional dimensionality reduction and clustering of epithelial cells, we established a multi-algorithm pipeline integrating monocle2, Slingshot and CytoTRACE to evaluate the differentiation trajectories and stemness dynamics across six AEG malignant epithelial cell clusters. Monocle2-based pseudotemporal reconstruction demonstrated that all epithelial cells were categorized into three distinct states, with a singular developmental progression originating from subgroup C2 (**Figures 4A, B**), which closely aligned with the results from Slingshot analysis (**Figure 4C**). Subsequently, to unravel the transcriptional changes associated with pseudotime, we analyzed and visualized the temporal DEGs expression patterns across six subgroups in the context of monocle2 pseudotime, and the DEGs in the initial state of pseudotime were then subjected to GO-BP analysis. As expected, the DEGs were significantly enriched in epithelial to mesenchymal transition, Wnt signaling pathway and stem cell differentiation pathways (**Figure 4D**). CytoTRACE analysis revealed that subgroup C2 showed higher CytoTRACE scores, indicating greater differentiation potential, while subgroup C0 exhibited the lowest scores, suggesting a possible terminal stage of differentiation (**Figures 4E, F**).

**Figure 5:**
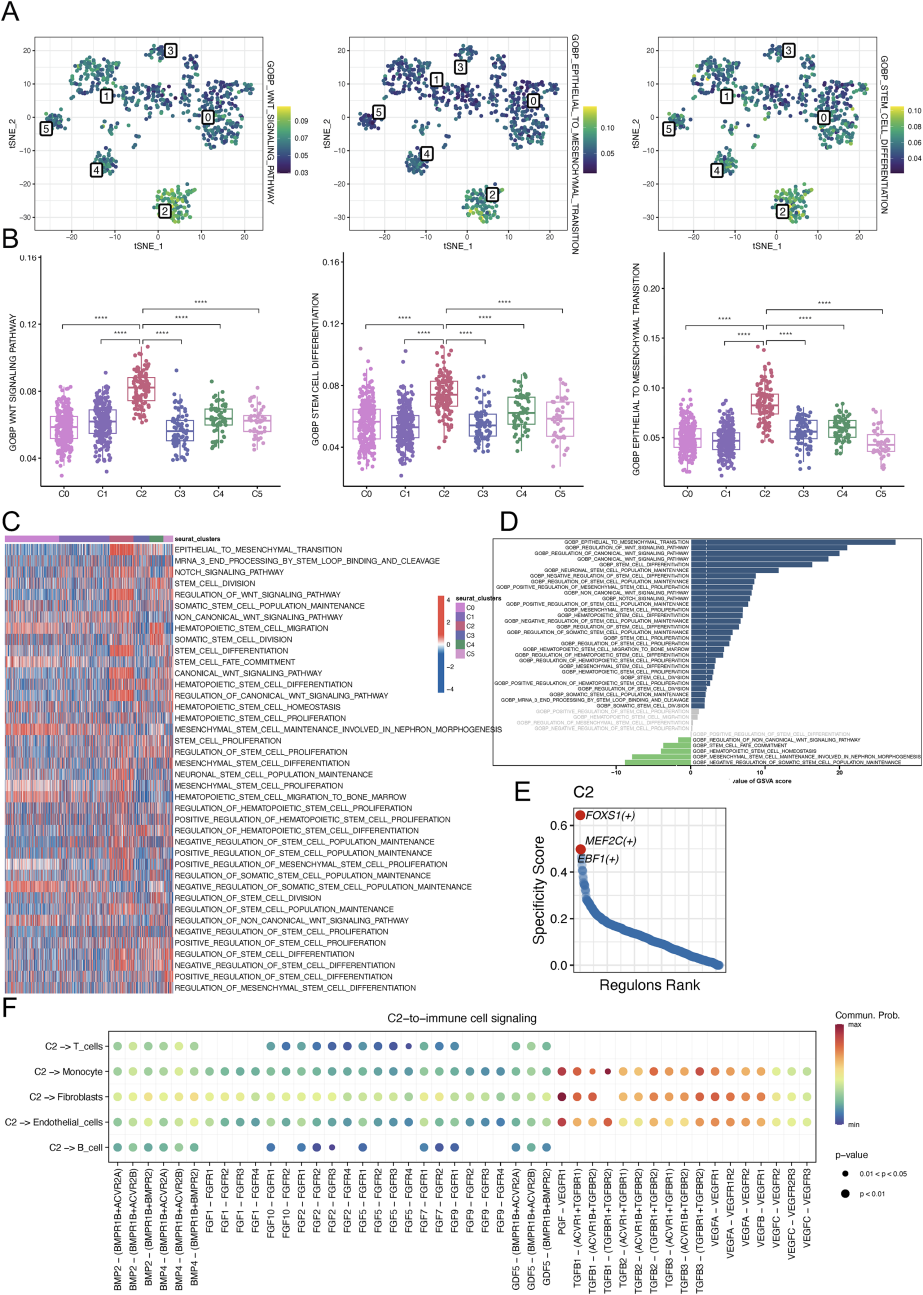
Stemness−related pathway enrichments and transcription factor networks analyses. **(A)** tSNE plot depicting the WNT signaling, epithelial to mesenchymal transition and stem cell differentiation scores of AUCell analysis among cell subsets. **(B)** Box plots for the WNT signaling, epithelial to mesenchymal transition and stem cell differentiation scores of AUCell analysis among cell subsets. **(C)** Heatmap showing the gsva scores of GO-BP stemness-related gene sets in different AEG malignant epithelial cell populations. **(D)** GSEA enrichment of stemness-related gene sets between C2 versus other epithelial cell clusters. **(E)** Transcription factor network identified FOXS1, MEF2C and EBF1 were the top 3 regulons. **(F)** CellChat bubble plot showing ligand−receptor communication probabilities from the putative stem−like cluster (C2) to immune cell types (B cell, Endothelial cells, Fibroblasts, Monocyte and T cells). Color intensity represents communication probability; dot size represents p−value. ****p < 0.0001. tSNE, t-distributed Stochastic Neighbor Embedding; GO-BP, Gene Ontology Biological Processes; AEG, Adenocarcinoma of esophagogastric junction; GSEA, Gene Set Enrichment Analysis.

To functionally delineate the stemness attributes, we performed integrative AUCell pathway enrichment profiling coupled with GSVA/GSEA analysis across cellular subpopulations. The C2 epithelial population showed significantly higher WNT signaling, EMT, and stem cell differentiation AUC scores than other subpopulations, indicating elevated invasive potential (**Figures 5A, B**). Meanwhile, GSVA was implemented with collected stemness signature gene sets, with results visualized via heatmap (**Figure 5C**). The heatmap revealed that most of the curated stemness gene sets were highly enriched in C2 subgroup. Additionally, by employing GSEA analysis using these stemness gene sets, significant enrichments of these gene sets, especially EMT, WNT pathways, were also observed in C2 subgroup compared to other epithelial subpopulations (**Figure 5D**).

Integration of monocle2, Slingshot and CytoTRACE analyses revealed the pivotal role of malignant epithelial C2 subpopulations in initiating tumoral evolution stage. GSVA/GSEA analyses further indicated high differentiation potential of C2 subgroup. Therefore, it was speculated that this subgroup could be regarded as a stem-like neoplastic subpopulation.

Furthermore, we conducted PySCENIC analysis on the transcription factor regulatory network of AEG malignant epithelial cells, and FOXS1, MEF2C and EBF1 were identified as the top three regulons in AEG malignant epithelial C2 cell clusters (**Figure 5E**). To investigate whether the putative CSC-like cluster (C2 cluster) could directly communicate with immune cells, we performed CellChat ligand−receptor interaction analysis. The top four pathways ranked by communication probability from C2 cluster to these immune cells were FGF, BMP, TGFb and VEGF (**Figure 5F**). Notably, TGF−β and VEGF are canonical drivers of immunosuppression. TGF−β inhibits CD8+ T cell cytotoxicity and induces regulatory T cell differentiation, while VEGF impedes T cell infiltration and dendritic cell activity. Although the FGF and BMP pathways are canonically linked to stemness regulation and developmental processes, they also contribute to the remodeling of the tumor microenvironment. These data provide direct ligand−receptor support that the C2 subpopulation shapes an immunosuppressive niche, most prominently via the TGF−β axis.

### Identification of hub gene modules of AEG malignant epithelial C2 by hdWGCNA

**Figure 6:**
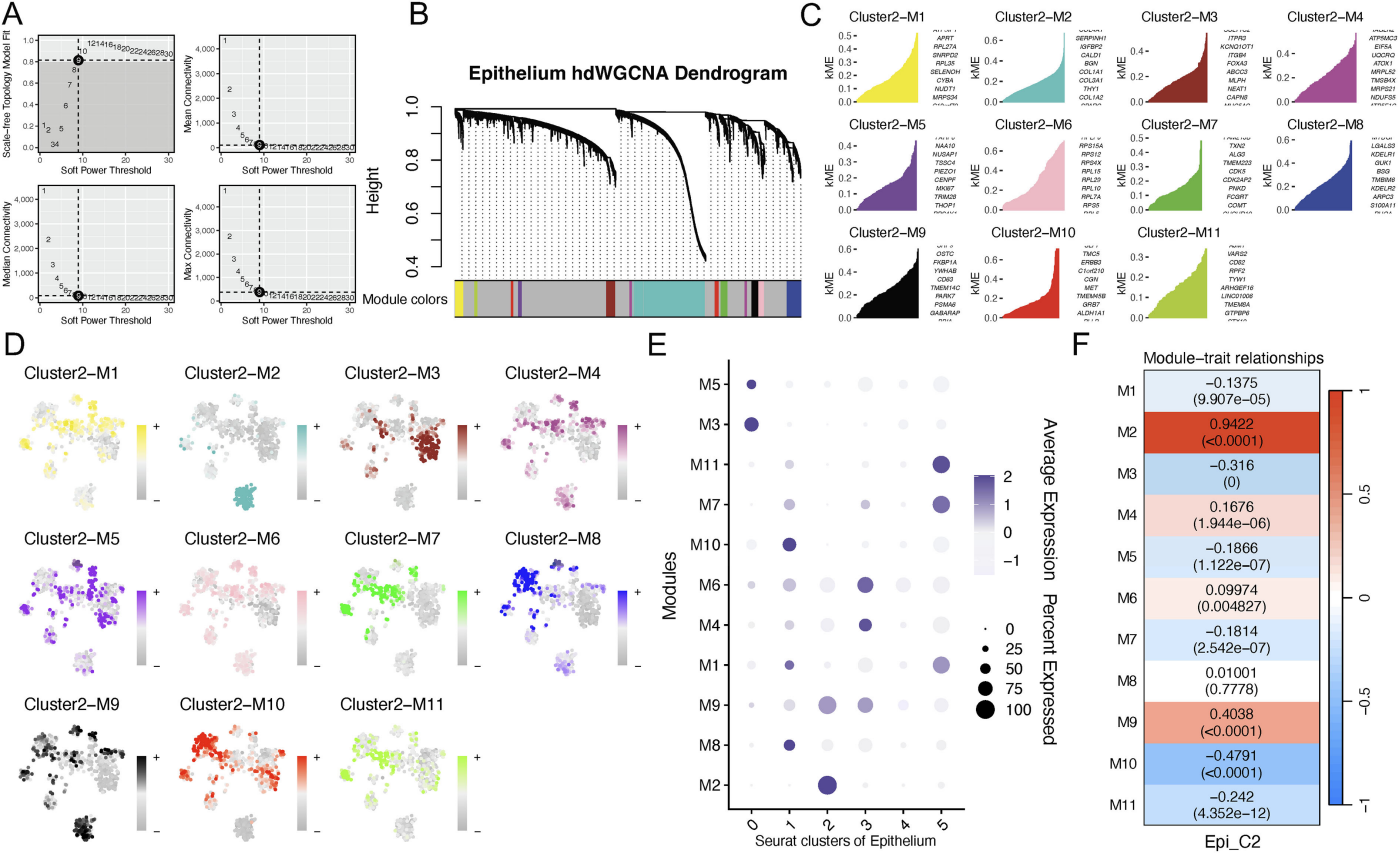
Malignant epithelial genes identification in AEG by hdWGCNA. **(A)** Soft threshold power selection. **(B)** Genes were divided into distinct modules by hierarchical clustering. **(C)** kME was calculated to identify genes with high connectivity levels in each module. **(D)** tSNE plot of AEG malignant epithelial cells with module scores. **(E)** Dot plot illustrates the expression profiles of diverse modules across distinct groups. **(F)** Heatmap depicting the correlation of MEs and AEG malignant epithelial cluster. hdWGCNA, High-dimensional weighted gene co-expression network analysis; tSNE, t-distributed Stochastic Neighbor Embedding; AEG, Adenocarcinoma of esophagogastric junction; MEs, module eigengenes.

Through hdWGCNA analysis of scRNA-seq data, the gene co-expression network was constructed to identify hub gene modules in AEG malignant epithelial cells. The soft threshold was firstly screened to build a scale-free network, and β = 9 was determined as the most suitable value (**Figure 6A**). Intermodular connectivity was evaluated by analyzing the distinct genes within each module, and 11 non-gray modules were identified and denoted by distinct colors (**Figure 6B**). Then, we calculated the kME, which represents the eigengene-based gene connectivity (**Figure 6C**). Subsequently, module scores for AEG malignant epithelial cells clusters were quantified and visualized (**Figure 6D**). Module-cluster association mapping demonstrated that the turquoise (M2) and black (M9) modules were significantly enriched in cluster 2 (**Figures 6E, F**). These findings suggest that the turquoise (M2) and black (M9) gene modules show specifically increased expression in AEG malignant epithelial cluster 2 cells.

### Integrative identification of AEG epithelial C2 hub genes and prognostic model construction

**Figure 7:**
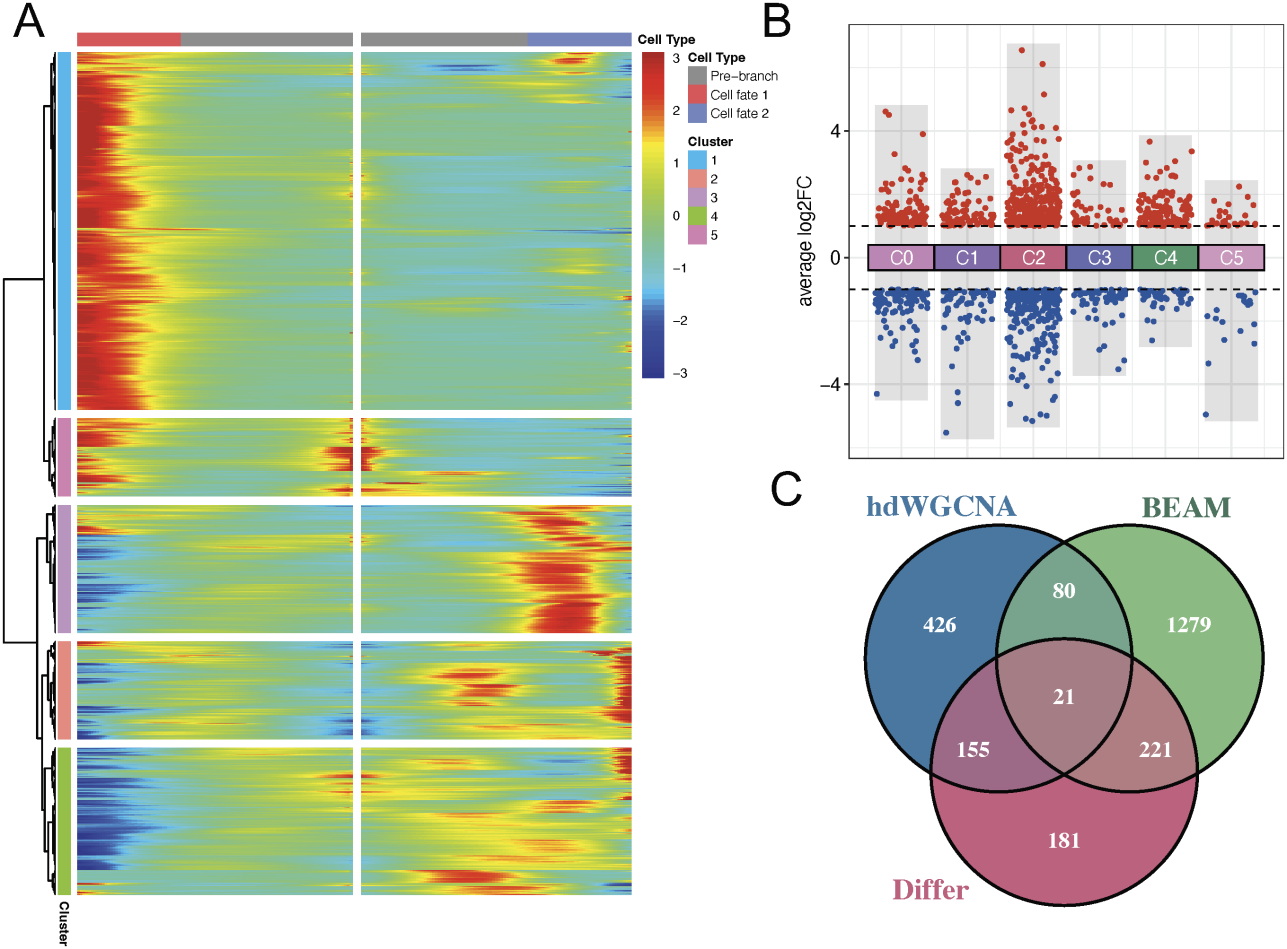
Integration of hdWGCNA, BEAM, and DEGs to identify hub genes. **(A)** The branched heatmap illustrating the expression dynamics of significant fate branch genes, with red denoting high expression levels and blue representing low expression levels. **(B)** DEGs in each cluster versus other AEG malignant epithelial cell clusters. **(C)** Venn plot illustrating the intersection of the hub genes of hdWGCNA, BEAM and DEGs of AEG malignant epithelial C2 cluster. DEGs, Differentially expressed genes; hdWGCNA, High-dimensional weighted gene co-expression network analysis; AEG, Adenocarcinoma of esophagogastric junction; BEAM, Branch Expression Analysis Modeling.

Three algorithms were applied for identifying AEG malignant epithelial C2 genes. Firstly, the BEAM algorithm following monocle analysis was employed to identify genes regulating branch node 1 (**Figure 4A**), and 1,601 genes were screened out with qval < 0.0001 (**Figure 7A**). Secondly, 578 DEGs between epithelial C2 and other clusters were determined using the criteria of an absolute log2FC > 1 and p-value < 0.05 (**Figure 7B**). Thirdly, 682 genes in turquoise (M2) and black (M9) modules with kME > 0.2 were extracted as hub module genes. By integrating the putative CSC-like epithelial C2 hub genes identified by above-mentioned three algorithms, a total of 21 genes were screened out as candidate CSCs genes of AEG (**Figure 7C**).

**Figure 8:**
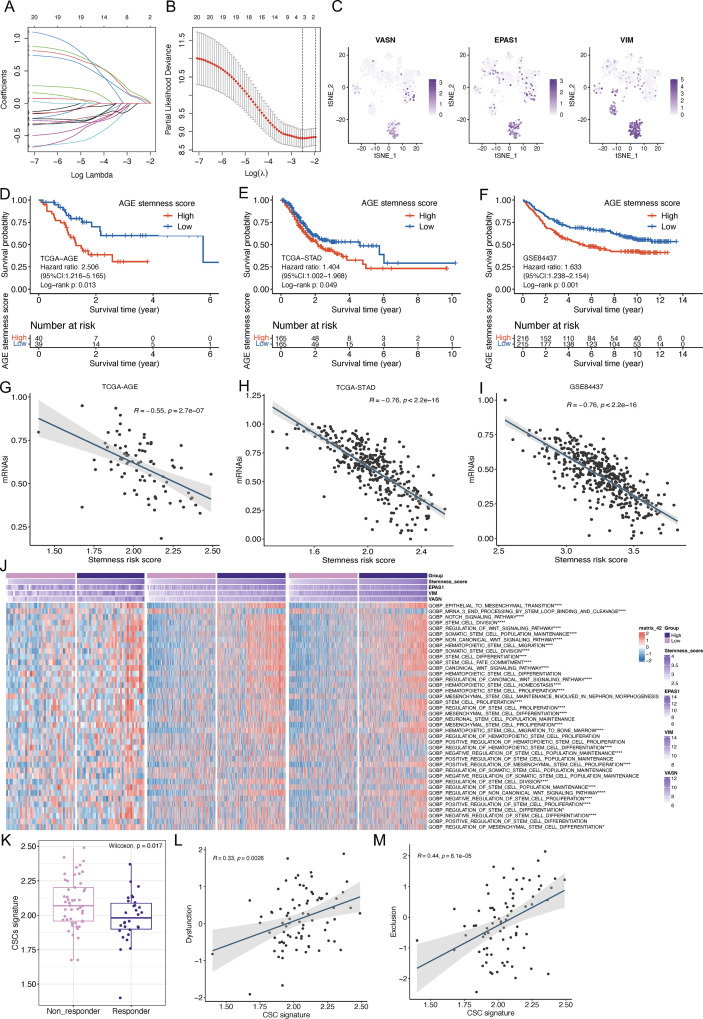
Prognostic model construction and validation. **(A, B)** Identification of prognostic stemness-related genes using LASSO Cox regression. **(A)** The optimal lambda screening by ten-fold cross-validation. **(B)** The optimal λ value facilitates the identification of key genes for prognostic model construction. **(C)** tSNE plot depicting the expressions of model genes among malignant epithelial cell subsets. **(D–F)** Kaplan-Meier curves for overall survival differences between high-stemness and low-stemness groups in TCGA-AEG **(D)**, TCGA-STAD **(E)** and GSE84437 **(F)** datasets. **(G–I)** The Spearman’s correlation analysis of stemness risk score with mRNAsi values in TCGA-AEG **(G)**, TCGA-STAD **(H)** and GSE84437 **(I)** datasets. **(J)** Heatmap showing the GSVA scores of GO-BP stemness-related gene sets in high-stemness and low-stemness groups in TCGA-AEG, TCGA-STAD and GSE84437 datasets. **(K)** Box plot revealed stemness risk score was higher in TIDE-predictted non−responsive patients. **(L, M)** The Spearman’s correlation analysis of stemness risk score with TIDE dysfunction **(L)** and exclusion **(M)** scores. *p < 0.05; ****p < 0.0001. tSNE, t-distributed Stochastic Neighbor Embedding; AEG, Adenocarcinoma of esophagogastric junction; STAD, Stomach Adenocarcinoma; GSVA, Gene Set Variation Analysis; GO-BP, Gene Ontology Biological Processes; TIDE, Tumor Immune Dysfunction and Exclusion.

Subsequently, for the convenience of clinical application, we applied LASSO Cox regression with an optimal lambda.min of 0.0804 for feature selection, and three core prognostic markers were ultimately identified for CSC-related prognostic model construction: VASN * 0.132 + VIM * 0.094 + EPAS1 * 0.077 (**Figures 8A, B**). The expression profiles of these three markers at single-cell resolution were depicted in **Figure 8C**. Based on the median risk score, the TCGA-AEG cohort (training) as well as TCGA-STAD and GSE84437 cohorts (validation) were stratified into high and low stemness-related groups. Kaplan-Meier survival curves revealed samples in high stemness-related group exhibited worse prognosis in TCGA-AEG (HR: 2.506, 95% CI: 1.216-5.165, Log-rank p = 0.013, **Figure 8D**), TCGA-STAD (HR: 1.404, 95% CI: 1.002-1.968, Log-rank p = 0.049, **Figure 8E**) and GSE84437 (HR: 1.633, 95% CI: 1.238-2.154, Log-rank p = 0.001, **Figure 8F**) cohorts. The model yielded a C-index of 0.646 (95% CI: 0.552–0.740) in the training cohort (TCGA-AEG), indicating modest discrimination for overall survival. When applied to the external validation cohorts, the C-index was 0.577 (95% CI: 0.538-0.615) in GSE84437 and 0.566 (95% CI: 0.514-0.617) in TCGA-STAD. The model’s performance in estimating AEG stemness was further evaluated via correlation analysis with mRNAsi index, which demonstrated a highly negative correlation with mRNAsi in all cohorts (**Figures 8G–I**). Additionally, correlation comparison analysis with the previously published four-gene signature in three cohorts revealed our three-gene model exhibited significant higher correlations with mRNAsi (**Supplementary Figure 1**), suggesting the enhanced capacity and superior clinical usability (lower computational complexity) of our model in reflecting AEG stemness. Moreover, we employed the GSVA method to examine differences in stemness-associated pathway scores between high and low-stemness risk groups. As visualized in the heatmap, stepwise increases of stemness-related risk scores positively correlated with concomitantly elevated stemness-related pathway scores in all three cohorts, and predictably, significant enrichment of these gene sets was observed in the high-risk subgroup (Wilcoxon test, **Figure 8J**). Furthermore, AEG patients predicted to be non−responsive by TIDE algorithm exhibited significantly higher CSC-related risk scores (Wilcoxon test, **Figure 8K**), and a significant positive correlation was also observed between the stemness score and TIDE dysfunction (**Figure 8L**) and exclusion (**Figure 8M**) scores.

### CSCs marker VASN could be regarded as a prognostic marker for AEG patients

**Figure 9:**
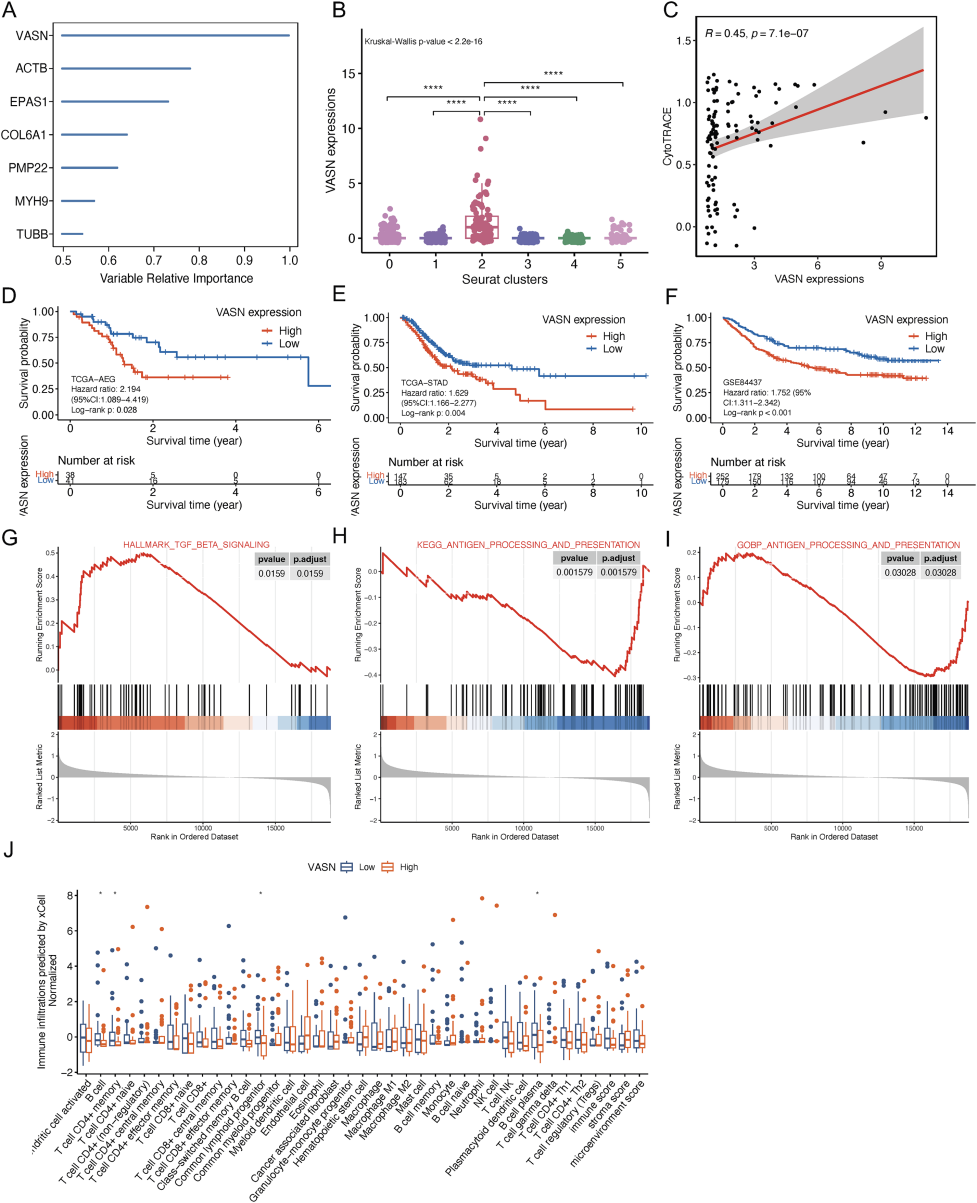
VASN is a candidate stemness−associated gene with prognostic significance. **(A)** Random survival forest analysis determined the prognostic importance of candidate stemness-related genes. **(B)** Box plots revealed VASN was highly expressed in malignant epithelial C2 cluster. **(C)** The Spearman’s correlation analysis of VASN expressions with CytoTRACE scores. **(D–F)** Kaplan-Meier curves for overall survival differences between high and low VASN expression groups in TCGA-AEG **(D)**, TCGA-STAD **(E)** and GSE84437 **(F)** datasets. **(G–I)** GSEA analysis of TGF-β **(G)** and antigen processing and presentation **(H, I)** gene sets between high and low VASN-expression groups. **(J)** Boxplots showing the infiltration differences between VASN−high and VASN−low groups. *p < 0.05; ****p < 0.0001. GSEA, Gene set enrichment analysis.

The “rfsrc” function from the randomForestSRC R package (version 2.9.3) was employed on TCGA-AEG cohort to robustly assess the relative importance of each candidate CSCs gene, and VASN was identified as the most important feature (**Figure 9A**). Notably, VASN exhibited the highest coefficient (0.132) in our CSC-related AEG prognostic model, and was also significantly increased in malignant epithelial C2 subgroup (**Figure 9B** and 8C) and positively correlated with CytoTRACE score (Spearman’s cor = 0.45, p = 7.1-e07) (**Figure 9C**). Moreover, Kaplan-Meier survival curves revealed high VASN expression correlated with worse prognosis in TCGA-AEG (HR: 2.194, 95% CI: 1.089-4.419, Log-rank p = 0.028, **Figure 9D**), TCGA-STAD (HR: 1.629, 95% CI: 1.166-2.277, Log-rank p = 0.004 **Figure 9E**) and GSE84437 (HR: 1.752, 95% CI: 1.311-2.342, Log-rank p < 0.001, **Figure 9F**) cohorts. In addition, we also observed that the HALLMARK_TGF_BETA_SIGNALING gene set (**Figure 9G**) was significantly enriched in the VASN-high group, while the KEGG_ANTIGEN_PROCESSING_AND_PRESENTATION (**Figure 9H**) and GOBP_ANTIGEN_PROCESSING_AND_PRESENTATION (**Figure 9I**) gene sets were significantly enriched in VASN-low group (grouped by median VASN expression). To evaluate the impact of VASN expression on the immune landscape, we performed xCell analysis. Patients were stratified into VASN−high and VASN−low groups by median VASN expression. Compared to VASN−low group, VASN−high group exhibited significantly lower infiltration scores for B cells, CD4+ memory T cells, common lymphoid progenitors, and plasma cells (all p < 0.05, Wilcoxon tests). CD8+ T cell infiltration showed a decreasing trend in the VASN−high group, although this did not reach statistical significance (p = 0.065) (**Figure 9J**). Collectively, these findings suggest that VASN−high AEG tumors are characterized by active TGF−β signaling, impaired antigen presentation and a broad suppression of adaptive immune cells, supporting a model in which VASN contributes to an immune−evasive microenvironment that underlies unfavorable clinical outcomes in AEG.

### VASN knockdown reduces CSC characteristics of AEG cells

**Figure 10:**
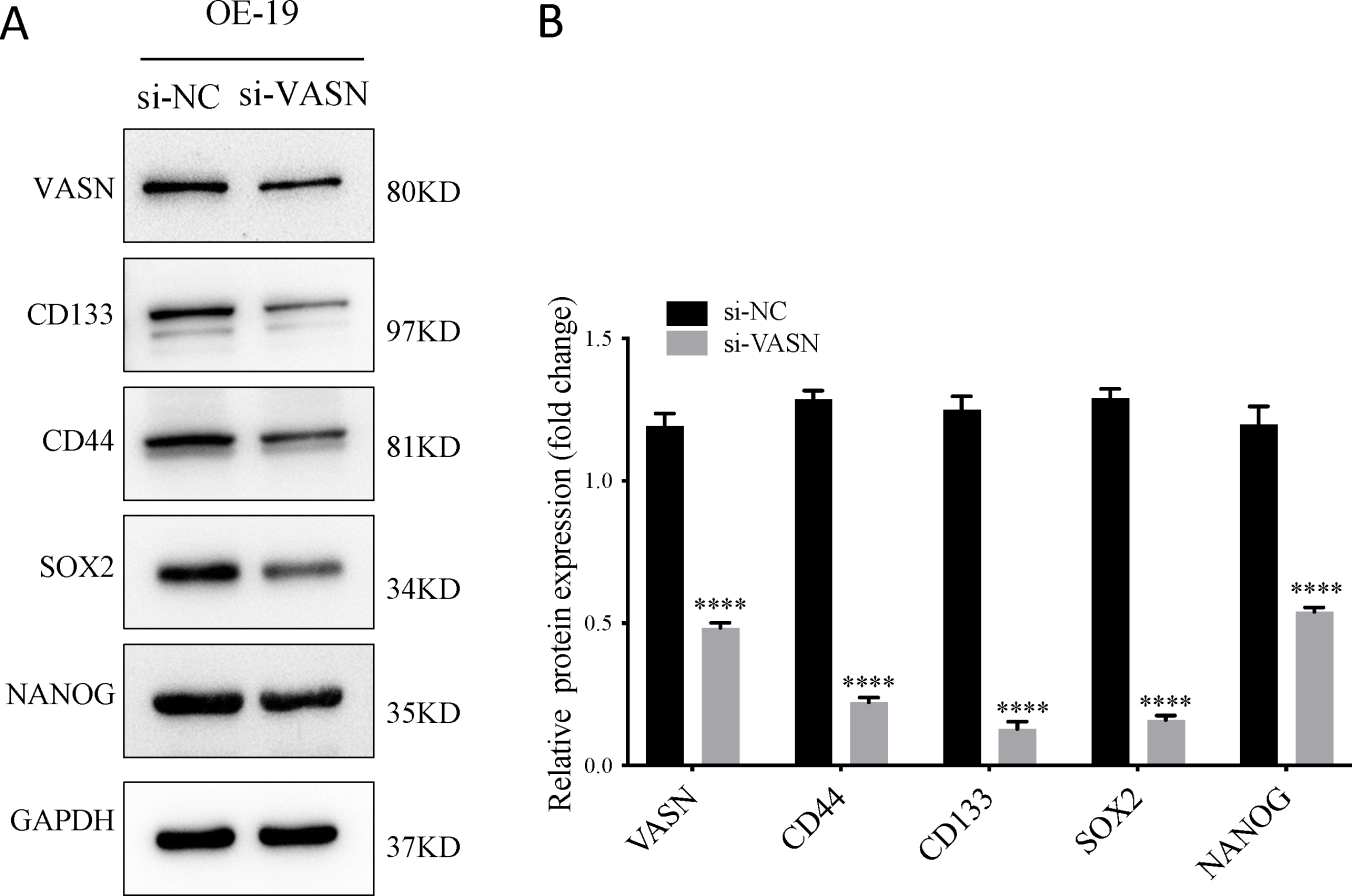
**(A, B)** OE19 cells were transfected with si-VASN and si-NC, and the expression levels of VASN, CD133, CD44, SOX2 and Nanog were evaluated by western blotting. ****p < 0.0001.

To investigate the impact of VASN on the CSC characteristics of AEG cells, the OE19 cell line derived from an AEG patient (36) were transfected with VASN-targeting small interfering RNA (si-VASN), while a scrambled negative control siRNA (si-NC) was used as control. Accumulating evidence has linked CD44 and CD133 to CSC phenotype in AEG (7). In our study, the protein expressions of CSC markers CD44 and CD133 were examined by WB, and results indicated CD44 and CD133 protein levels were decreased after si-VASN transfections (**Figures 10A, B**). Taken together, our findings indicate that VASN is closely linked to CSCs-related properties in AEG and may exert a pivotal influence on AEG tumorigenesis and progression.

### VASN knockdown inhibits EMT process in AEG cells

**Figure 11:**
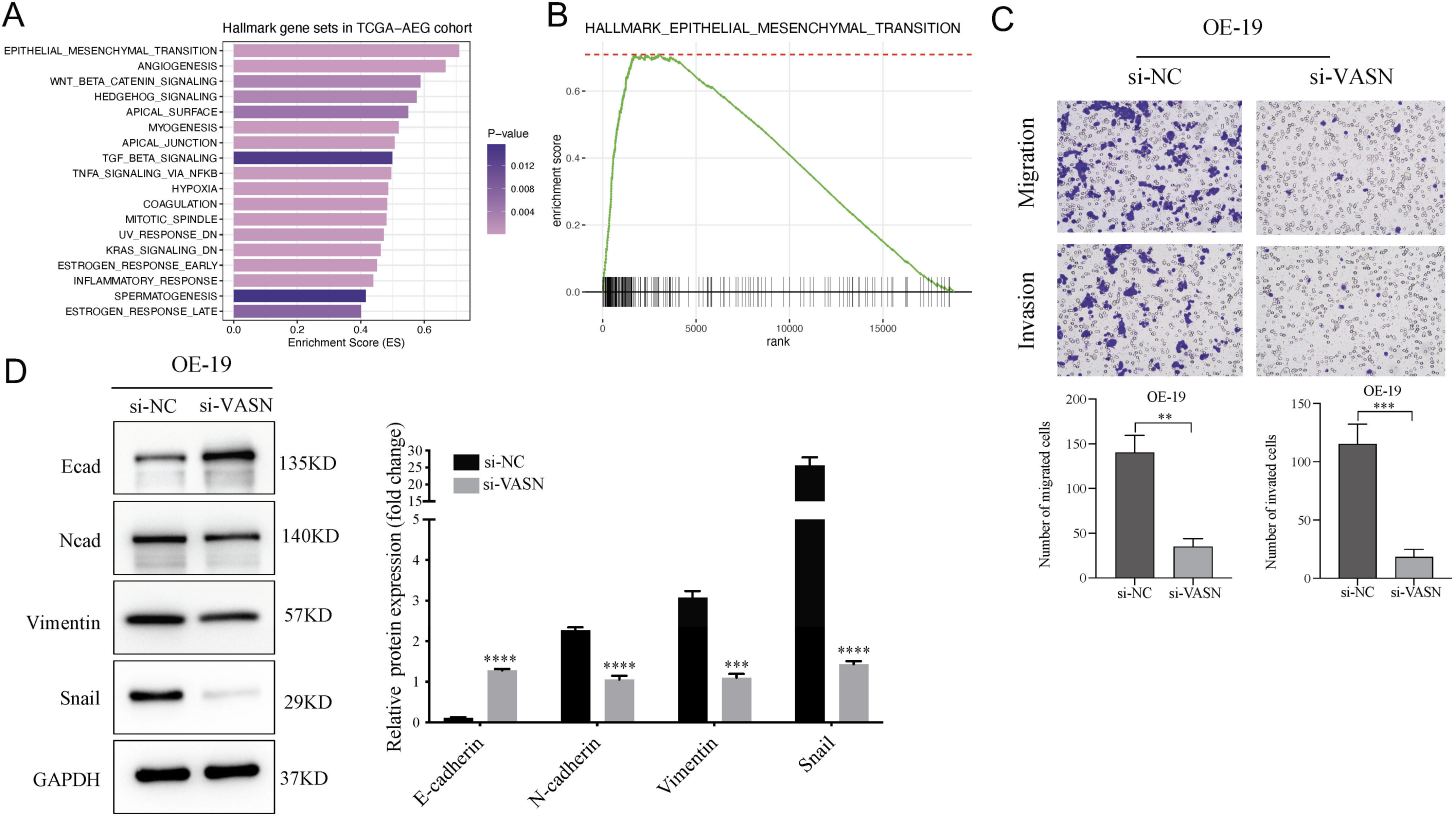
GSEA analysis and VASN knockdown suppresses EMT traits in AEG cells **(A)** The bar chart displays the GSEA results using the hallmark gene sets, with bar lengths indicating enrichment scores and colors representing p-values. **(B)** GSEA analysis of EMT gene set between high and low VASN groups. **(C)** Transwell assays revealed that VASN knockdown significantly reduced the migration and invasion abilities of AEG cancer cells. **(D)** OE19 cells were transfected with si-VASN and si-NC, and the expression levels of Ecad, Ncad, Vimentin and Snail were evaluated by western blotting. **p < 0.01; ***p < 0.001; ****p < 0.0001. GSEA, Gene set enrichment analysis.

GSEA analysis was conducted on hallmark gene sets on TCGA-AEG cohort using the median VASN expression level as the cutoff. The results indicated that the EMT pathway was significantly enriched in the high VASN expression group, with the enrichment score ranking first among all Hallmark pathways (**Figures 11A, B**). Given that accumulating evidence suggests EMT activation confers stem-like traits to tumor cells (7), we further functionally investigate the impact of VASN on EMT, and migration and invasion assays were performed. The Transwell assay in **Figure 11C** showed that VASN knockdown significantly diminished migratory and invasive abilities in OE19 cells compared to those treated with si-NC transfections. Moreover, western blotting demonstrated that EMT was inhibited in VASN-knockdown cells, as evidenced by reduced expression of mesenchymal markers (Snail, Vimentin, N-Cadherin) and elevated expression of epithelial marker E-cadherin (**Figure 11D**).

### Wnt/β-catenin signaling was involved in VASN-promoted CSC characteristics in AEG

**Figure 12:**
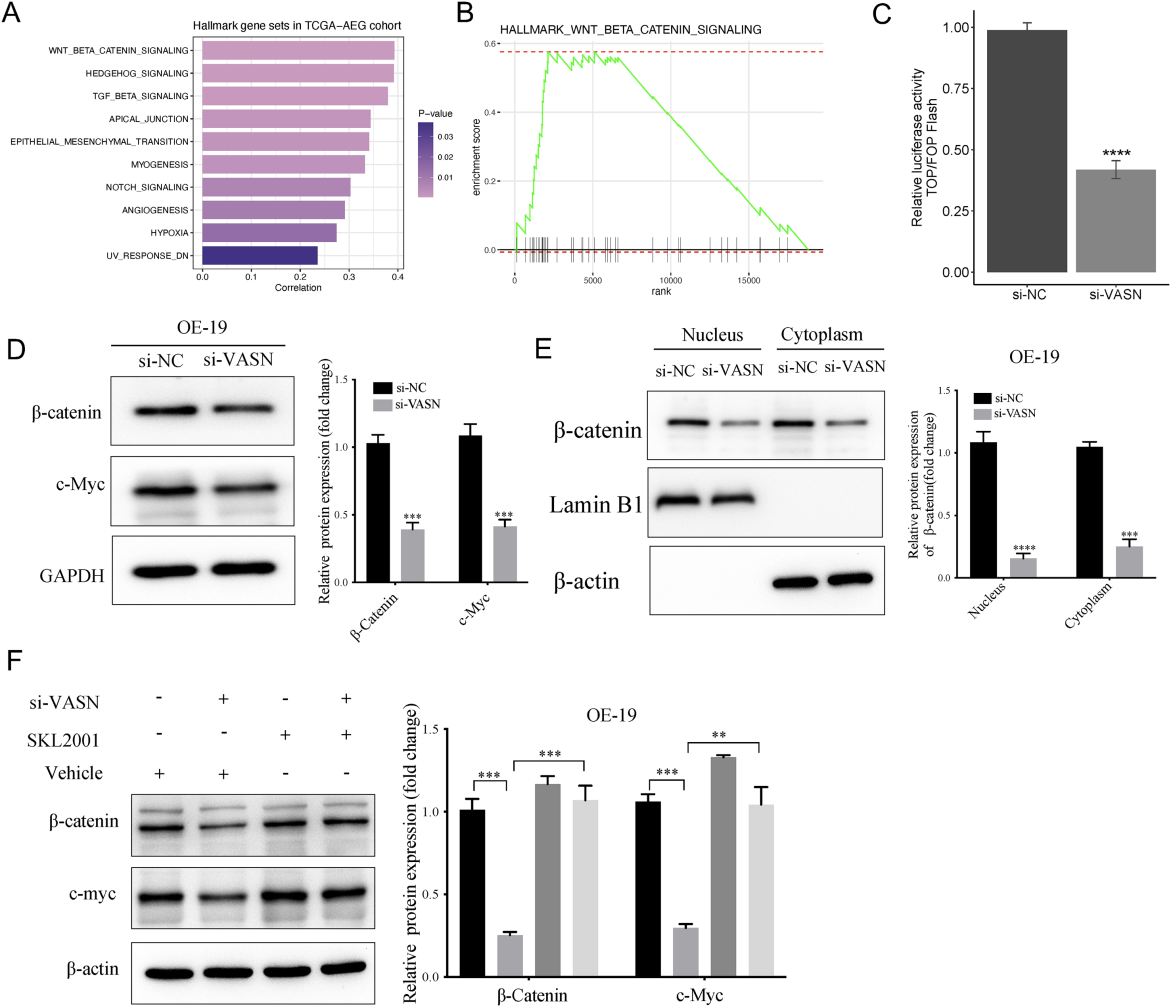
VASN knockdown correlated with decreased Wnt/β−catenin signaling **(A)** The bar chart displays the Gene set enrichment analysis of stemness gene sets, with bar lengths indicating enrichment scores and colors representing p-values. **(B)** Gene set enrichment analysis of wnt/beta-catenin gene sets between high and low VASN groups. **(C)** TOP/FOP-flash assays demonstrating that VASN knockdown significantly nhibits β-catenin-mediated TCF/LEF transcriptional activity in OE19 cells. **(D)** OE19 cells were transfected with si-VASN and si-NC, and the expression levels of β-catenin and c-Myc were evaluated by western blotting. **(E)** OE19 cells were transfected with si-VASN and si-NC, and the expression levels of nuclear and cytoplasmic β-catenin were evaluated by western blotting. **(F)** Western blotting was employed to assess the reversal efficacy of SKL2001 on the expressions of key proteins of Wnt/β-catenin signaling pathway in si-VASN and si-NC transfected OE19 cells. **p < 0.01; ***p < 0.001; ****p < 0.0001.

To further elucidate the potential molecular mechanisms of VASN in AEG, we initiated our investigation with Hallmark GSEA and GSVA analyses on TCGA-AEG bulk RNA-Seq data to identify enriched pathways. Through GSVA pathway quantification analysis, we observed the Wnt/β-catenin pathway exhibited maximal positive correlation with VASN expression levels (**Figure 12A**), GSEA also revealed Wnt/β-catenin pathway was highly enriched in VASN-high group (**Figure 12B**). Overall, the above bioinformatic results indicated that VASN might regulate Wnt/β-catenin pathway activation in AEG cells, which motivated us to investigate the role of VASN in Wnt/β-catenin signaling axis. We firstly performed TOP/FOP-Flash luciferase reporter assays to assess the β-catenin-mediated TCF transcriptional activity after VASN knockdown, results showed that compared with the control group, VASN knockdown significantly suppressed β-catenin-mediated TCF/LEF transcriptional activity in OE19 cells (**Figure 12C**). Additionally, Western blotting analysis revealed the protein expression levels of key genes (β-catenin and c-Myc) in Wnt/β-catenin signaling were inhibited after VASN knockdown (**Figure 12D**). Nuclear-cytoplasmic fractionation assays revealed that VASN knockdown significantly reduced the β-catenin levels in both cytoplasm and nuclear of OE19 cells (**Figure 12E**). Importantly, we further treated VASN knockdown OE19 cells with SKL2001 (an agonist of Wnt/β-catenin pathway), and observed that SKL2001 application significantly reversed the down-regulation of β-catenin as well as its downstream target gene c-Myc induced by VASN knockdown in OE19 cells (**Figure 12F**). Collectively, these results indicate that VASN knockdown correlates with reduced Wnt/β−catenin activity and decreased stemness−associated properties. However, causality remains to be established through rescue and *in vivo* experiments.

## Discussion

Technological advancements in bioinformatics, especially scRNA-seq, have enabled researchers to perform high-resolution gene expression profiling at single-cell level within the tumor microenvironment, uncovering the complex cellular diversity inherent to neoplastic tissues (38). AEG is a highly aggressive and molecularly heterogeneous malignancy, which harbors distinct molecular features compared with esophageal and gastric adenocarcinoma, highlighting the critical need to characterize molecular signatures with therapeutic target potential. However, the optimal therapeutic strategies for AEG remain controversial (39, 40). AEG is characterized by adverse clinical prognosis and pronounced drug resistance (3). Even for AEG patients treated with curative resection, approximately 55-60% would experience recurrence within 5 years, and the median OS for patients with metastatic or recurrent disease is only 11–12 months (41). CSCs serve as key drivers in AEG progression and contribute to its heterogeneity, and accumulating evidence suggests that stemness-associated programs may also influence tumor microenvironment organization. Thus, CSC-based innovative therapeutic strategies are critically needed. Our study integrated scRNA-seq, bulk RNA-seq and machine learning algorithms to unveil the heterogeneity of malignant epithelial cells, the C2 malignant epithelial cells positioned at the origin of pseudotime trajectories (monocle2), which also exhibited elevated CytoTRACE scores and high enrichments of EMT and Wnt signaling gene sets (GSEA/GSVA), were tentatively identified as the putative CSC-like subpopulation in AEG. Further, we developed a CSC-signature based on CSCs makers as a predictive model for AEG patient outcomes. Collectively, these results highlight the biological significance of stem-like epithelial programs in AEG progression.

Subsequently, through differential expression analysis, branch expression modeling and high-dimensional weighted gene co-expression network analysis, we explored the core gene sets of putative CSC-like cells subpopulation across multiple dimensions. By integrating bulk transcriptomic analysis with LASSO Cox regression and random survival forest machine learning algorithms in TCGA-AEG cohort, we identified VASN as the core gene of the CSC subpopulation in AEG. High VASN expression correlated with adverse prognosis in AEG patients, and VASN knockdown significantly suppressed the CSC phenotypes in AEG cells. Notably, EMT phenotypes contribute to CSCs enrichment and therapeutic resistance, and regulators involved in EMT and CSCs maintenance have emerged as promising targets (6, 42). Our results demonstrated that silencing VASN effectively abolished migration, invasion, and EMT-associated molecular expression, and markedly downregulated the CSCs markers CD44 and CD133. Collectively, our findings position VASN as a candidate regulator of stemness and EMT phenotypes in AEG.

Beyond its role in tumor propagation, emerging evidence indicates that cancer stem-like cells actively participate in shaping the tumor immune microenvironment. Stemness-associated pathways, particularly the aberrant activation of Wnt/β-catenin pathway and EMT-related programs, are mechanistically linked to immune exclusion and immunosuppressive microenvironment formation, which in turn restricts cytotoxic T-cell infiltrations across multiple solid tumors (43–45). In this context, the marked activation of Wnt/β-catenin signaling and stemness-related programs in the C2 subpopulation suggests that this epithelial compartment might contribute to immunological niche remodeling in AEG. Our CellChat analysis provides direct evidence that the putative CSC−like subpopulation communicates with multiple immune cell types via the TGF−β and VEGF pathways, both of which are known to suppress antitumor immunity (46, 47). Although FGF and BMP signaling also exhibited elevated communication probabilities, their established roles in stemness maintenance suggest potential indirect modulation of the immune landscape (48, 49). This might account for the poorer response to immune checkpoint inhibitors, as predicted by TIDE analysis. Our study also reveals that VASN expression is closely associated with an immunosuppressive tumor microenvironment in AEG. Specifically, GSEA demonstrated that the TGF-β signaling pathway was significantly enriched in VASN-high AEG patients, while the KEGG and GO-BP antigen processing and presentation pathways were significantly enriched in VASN-low group. Our xCell analysis revealed that VASN−high tumors exhibited significantly lower levels of B cells, CD4+ memory T cells, common lymphoid progenitors, and plasma cells, along with a trend toward reduced CD8+ T cells (p = 0.065). Although the CD8+ T cell reduction did not reach significance, the consistent directional trend supports a potential link between VASN expression and impaired cytotoxic T cell infiltration, warranting validation in larger AEG cohorts. TGF-β is a pleiotropic cytokine that suppresses antitumor immunity by inhibiting CD8+ T cell activation, promoting regulatory T cell differentiation, and impairing dendritic cell function (50–52). Correspondingly, the xCell-based immune infiltration analysis revealed that VASN-high AEG tumors exhibited significantly lower infiltrations of CD8+ T cells, CD4+ memory T cells, B cells and plasma cells compared to VASN-low AEG tumors, indicating a widespread adaptive immune deficiency. Collectively, these preliminary bioinformatic findings suggest that VASN might contribute to the formation of an immune-suppressive niche in AEG, potentially involving TGF-β signaling and reduced CD8+ T cell recruitment (51, 53). Further functional studies are required to delineate the causal relationship between VASN-driven stemness and these immune features.

Emerging studies have highlighted the pivotal role of VASN in modulating tumor stemness. High VASN expression indicates an adverse prognosis in gastric cancer (54), breast cancer (55), glioma (56), colorectal cancer (57), and glioma (58). While the significant roles of VASN in various malignancies have been elucidated, its potential regulatory mechanisms are divergent. Helicobacter pylori infection induces HIF-1α to upregulate VASN, which promotes gastric carcinogenesis by regulating COL4A1-PI3K/AKT pathway to enhance gastric epithelial cell malignancy (54). Kang et al. and Liang et al. demonstrated that VASN drives colorectal cancer proliferation, drug resistance and pulmonary metastasis, mainly by coregulation of NOTCH1 and MAPK pathways (57) as well as YAP/TAZ and AKT signaling pathways (59). Wan et al. reported VASN contributes to hepatocellular carcinoma carcinogenesis via STAT3 signaling pathway (60). In glioma, Liang et al. reported VASN facilitates glioma progression and angiogenesis, and sustains glioma CSC traits via the activation of the STAT3 pathway and suppression of the NOTCH pathway (58); Man et al. reported that under hypoxic conditions, Vasorin (VASN-encoded cell surface protein) was more robustly induced, and the Notch signaling was further amplified for the maintenance of glioma stem cell populations in hypoxic niches, proposing the strategy of targeting Vasorin to deplete the hypoxic CSCs in glioma (61). Zhao et al. demonstrated that soluble VASN promoted tumor progression through CD71-mediated endocytosis and STAT3 activation across cancerous, endothelial and T cells, proposed the sVASN-CD71 axis as a novel therapeutic target (62). Growing evidence highlights the critical role of Wnt/β-catenin signaling on tumor stemness maintenance in gastric and esophageal cancers (63). In our study, we for the first time identified VASN as a novel biomarker of stem-like epithelial subpopulations in AEG, and through integrated bulk RNA-seq deconvolution with functional validations, we further found that VASN knockdown correlated with decreased Wnt/β−catenin signaling and diminished stemness traits, suggesting a potential link between VASN and this pathway. Previous study from Smith et al. also demonstrated that VASN regulates osteoclast-osteoblast crosstalk via Wnt/β-catenin signaling pathway, positioning VASN as a therapeutic target for osteoblast-osteoclast imbalance bone disorders (64). Thus, we propose that VASN-mediated antagonism of β-catenin activity might exert anti-tumor effects by disrupting stemness maintenance in AEG, and the identified C2-VASN axis might represent a tumor-intrinsic mechanism that modulates immune landscape organization in AEG, providing a conceptual link between epithelial stemness and organ-specific immunological niche dynamics.

Several limitations should be acknowledged. Firstly, our single-cell transcriptomic analysis was performed on only two AEG samples. The small sample size cannot comprehensively capture the intratumoral and intertumoral heterogeneity of AEG, thereby raising concerns about the reproducibility and generalizability. Hence, the conclusions derived from the scRNA-seq are exploratory, and definitive conclusions necessitate independent validation in larger, multi-sample scRNA-seq cohorts. Nevertheless, despite the limited sample size of the scRNA−seq analysis, the subsequent bulk RNA−seq validations supported key findings such as the prognostic value of VASN and the enrichment of relevant pathways, thereby partially alleviating concerns about generalizability. Secondly, due to the relatively limited sample size of the AEG training cohort (n=79) and the heterogeneity between training and validation cohorts (the latter included non-AEG gastric cancer samples), the model’s prognostic predictive performance is limited. This observation also indirectly supports the notion that AEG should be considered a distinct clinicopathological entity, and further validations are required in larger AEG-specific cohorts (65). Third, interpretation of VASN in Wnt/β−catenin signaling is limited by the absence of rescue experiments, validation in other AEG cell lines and *in vivo* models. Future functional and animal studies are needed for definitive mechanistic confirmation.

## Conclusions

We conducted an exploratory scRNA-seq analysis to characterize the putative CSC-like epithelial cells in AEG and identified candidate hub regulators pivotal for AEG stemness maintenance. Among these, VASN was further screened out as a candidate marker. High VASN expression was associated with an unfavorable prognosis in AEG patients, and VASN knockdown not only suppressed AEG cell migration and invasion abilities but also acted as a candidate modulator of AEG cell stemness by suppressing the Wnt/β-catenin signaling pathway. These results suggest VASN as a prognostic biomarker and may play a tumor-suppressive role in modulating AEG stemness. Further investigations into the molecular mechanisms underlying VASN’s interactions with its targets in regulating cancer cell biology are warranted. Collectively, our findings provide preliminary evidence for the potential involvement of VASN in AEG CSCs-like characteristics, and provide new research directions for CSC-targeted therapy in AEG.

## Data Availability

The original contributions presented in the study are included in the article/**Supplementary Material**. Further inquiries can be directed to the corresponding authors.

## Supplementary material

Supplementary Figure 1 The Spearman’s correlation analysis of the published stemness signature with mRNAsi values in TCGA-AEG **(A)**, TCGA-STAD **(B)** and GSE84437 **(C)** datasets.
