# Supplementary figures and images for: Integrated single-cell and bulk transcriptomic analyses reveal a stem-like epithelial subpopulation in adenocarcinoma of the esophagogastric junction and identify VASN as a novel regulator of tumor stemness
Source: Front Immunol. 2026 May 13;17:1817030. doi: 10.3389/fimmu.2026.1817030 (PMC13212525; doi:10.3389/fimmu.2026.1817030)

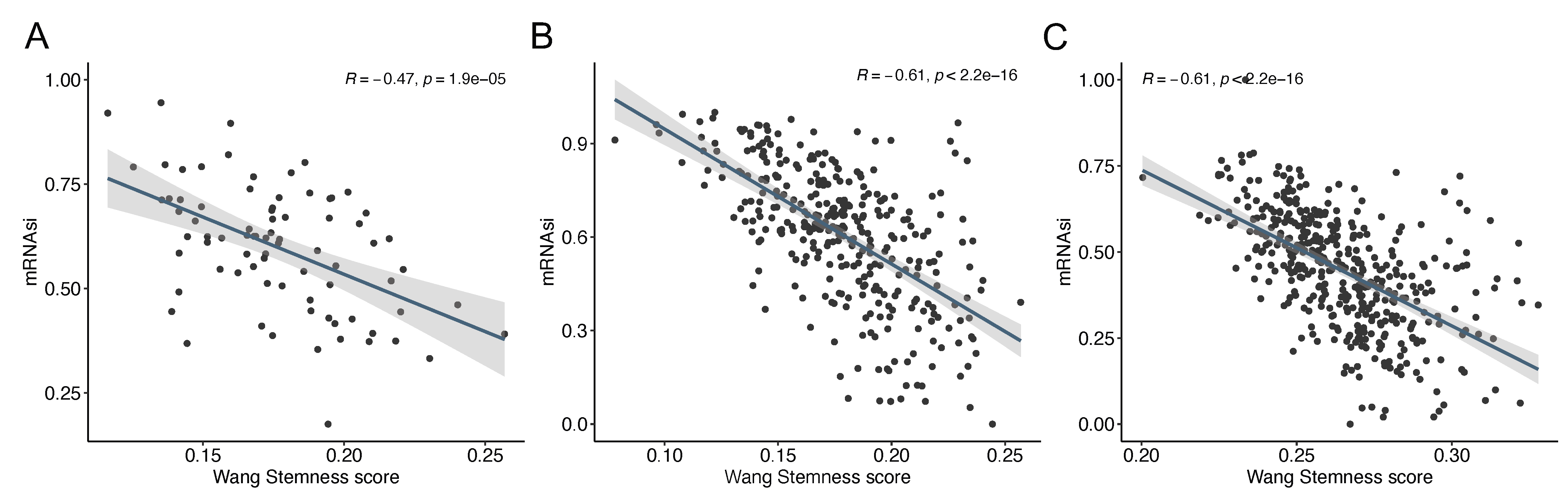

Supplement: Supplementary Figure 1 — The Spearman’s correlation analysis of the published stemness signature with mRNAsi values in TCGA-AEG (A), TCGA-STAD (B) and GSE84437 (C) datasets. [file Image1.tiff]
